# Hierarchical inhibition of mTORC1 by glucose starvation-triggered AXIN lysosomal translocation and by AMPK

**DOI:** 10.1093/lifemeta/load005

**Published:** 2023-03-01

**Authors:** Mengqi Li, Xiaoyan Wei, Jinye Xiong, Jin-Wei Feng, Chen-Song Zhang, Sheng-Cai Lin

**Affiliations:** State Key Laboratory of Cellular Stress Biology, School of Life Sciences, Faculty of Medicine and Life Sciences, Xiamen University, Xiamen, Fujian 361102, China; State Key Laboratory of Cellular Stress Biology, School of Life Sciences, Faculty of Medicine and Life Sciences, Xiamen University, Xiamen, Fujian 361102, China; State Key Laboratory of Cellular Stress Biology, School of Life Sciences, Faculty of Medicine and Life Sciences, Xiamen University, Xiamen, Fujian 361102, China; State Key Laboratory of Cellular Stress Biology, School of Life Sciences, Faculty of Medicine and Life Sciences, Xiamen University, Xiamen, Fujian 361102, China; State Key Laboratory of Cellular Stress Biology, School of Life Sciences, Faculty of Medicine and Life Sciences, Xiamen University, Xiamen, Fujian 361102, China; State Key Laboratory of Cellular Stress Biology, School of Life Sciences, Faculty of Medicine and Life Sciences, Xiamen University, Xiamen, Fujian 361102, China

**Keywords:** mTORC1, glucose sensing, AMPK

## Abstract

When glucose is replete, mammalian/mechanistic target of rapamycin complex 1 (mTORC1) is active and anchored to the lysosomal surface via the two GTPases, Ras-related GTPase (RAG) and Ras homolog enriched in brain (Rheb), which are regulated by Ragulator and tuberous sclerosis complex 2 (TSC2), respectively. When glucose is low, aldolase senses low fructose-1,6-bisphosphate level and promotes the translocation of AXIN−liver kinase B1 (LKB1) to the lysosomal surface, which leads to the activation of AMP-activated protein kinase (AMPK) and the inhibition of RAGs, sundering mTORC1 from the lysosome and causing its inactivation. AMPK can also inactivate mTORC1 by phosphorylating Raptor and TSC2. However, the hierarchy of AXIN- and AMPK-mediated inhibition of mTORC1 remains poorly defined. Here, we show that AXIN translocation does not require AMPK expression or activity. In glucose starvation conditions, knockout of AXIN extended the half-life of mTORC1 inhibition from 15 to 60 min, whereas knockout of AMPK only extended it to 30 min. RAGB^GTP^ (constitutively active RAGB) almost entirely blocked the lysosomal dissociation and inhibition of mTORC1 under glucose starvation, but it did not inhibit AMPK, indicating that under these conditions, it is AXIN lysosomal translocation that inhibits mTORC1, and it does so via inhibition of RAGs. 5-aminoimidazole-4-carboxamide ribonucleoside (AICAR), a mimetic of AMP, which activates both cytosolic AMPK and lysosomal AMPK, fully inhibited mTORC1 even when it is stably anchored to the lysosome by RAGB^GTP^, whereas glucose starvation mildly inhibited such anchored mTORC1. Together, we demonstrate that the lysosomal translocation of AXIN plays a primary role in glucose starvation-triggered inhibition of mTORC1 by inhibiting RAGs, and that AMPK activity inhibits mTORC1 through phosphorylating Raptor and TSC2, especially under severe stress.

## Introduction

Glucose is the most common carbon source for energy production and the biosynthesis of amino acids and lipids in most organisms. However, increasing evidence has demonstrated that glucose and its intermediary metabolites, once sensed by cellular sensors, can act as “messengers” to elicit a wide range of physiological changes. AMP-activated protein kinase (AMPK) and mammalian/mechanistic target of rapamycin complex 1 (mTORC1), exerting opposing roles to maintain metabolic homeostasis by promoting catabolism and anabolism respectively, are among the effectors of the sensing machineries [1]. It has been shown that glucose starvation-mediated activation of AMPK, as well as subsequent inhibition of mTORC1, occurs prior to the decline in the energy supply [2–6] that only occurs during prolonged starvation, contrary to the common belief that AMPK activation depends on an energy shortage [7, 8].

The glucose sensing that leads to AMPK activation takes place on the lysosomal surface, and it is the glycolytic intermediate fructose-1,6-bisphosphate (FBP) itself that is sensed, which is directly coupled to the regulation of AMPK and mTORC1 [6, 9–13]. The sensing of FBP is mediated by the lysosome-localized, vacuolar H^+^-ATPases (v-ATPase)-associated glycolytic enzyme aldolase [14], the very enzyme that cleaves FBP to phosphotrioses dihydroxyacetone phosphate (DHAP) and glyceraldehyde-3-phosphate (G3P). Thus, when glucose levels (and hence FBP) are low, an increased proportion of aldolase becomes FBP-unoccupied, which in turn blocks the endoplasmic reticulum (ER)-localized transient receptor potential V (TRPV) calcium channels, converting the low cellular-glucose signal to a low-calcium signal at the ER-lysosome contact [6, 15]. TRPVs then interact with v-ATPase in a manner inversely correlated to Ca^2+^ concentration, presumably causing a re-configuration of the aldolase-v-ATPase complex, resulting in inhibition of v-ATPase [15]. As a result, the intrinsically disordered scaffold protein AXIN utilizes v-ATPase and its associated Ragulator (comprised of 5 LAMTOR subunits, LAMTOR1-5) [16–18] as docking sites [19].

Along with these interactions, AXIN undergoes conformational changes [19, 20], tethering liver kinase B1 (LKB1), an upstream kinase of AMPK, to the lysosomal surface through its C-terminus for activating nearby AMPK [19, 21, 22]. On binding to the N-terminus of AXIN, Ragulator undergoes conformational changes during which its ability to release GTP from RAGC [one component of the small GTPase RAGs (RAGA to RAGD)] is inhibited [23, 24], triggering the switch of RAG heterodimers from an “on” state (RAGA^GTP^-RAGC^GDP^) to an “off” state (RAGA^GDP^-RAGC^GTP^) [24, 25], and thereby decreasing the binding affinity between RAG-mTORC1 [17, 19, 26–29]. Consequently, mTORC1 is dissociated from the lysosome, keeping it away from its allosteric activator, the lysosomal pool of Ras homolog enriched in brain (Rheb) GTPase [17, 30–33]. It has also been reported that reduced levels of dihydroxyacetone phosphate (DHAP), the product of FBP catabolism, also help switch off mTORC1 when absent from the binding site in aldolase [20, 34]. Through such mechanisms, glucose starvation switches off mTORC1 to decrease anabolic activities under glucose starvation [1]. Conversely, high glucose level maintains the localization of mTORC1 on the lysosomal surface, thus maintaining its activity.

Apart from the inverse coordination between mTORC1 and AMPK on the lysosome, the enzymatic activity of mTORC1 can be inhibited by AMPK after activation during glucose starvation [35, 36]. It has been shown that AMPK can phosphorylate and hence increase the activity of tuberous sclerosis complex 2 (TSC2) [37], which is a GTPase-activating protein (GAP) of Rheb [30]. AMPK also phosphorylates the Raptor subunit of mTORC1, rendering mTORC1 inactive [38]. Very recently, it was also shown that in yeast, AMPK phosphorylates Pib2, a Raptor-binding protein, to inhibit the activity of TORC1 [39]. In addition, AMPK phosphorylates and activates uncoordinated 51-like kinase 1 (ULK1) [40, 41], which in turns phosphorylates leucyl-tRNA synthetase (LARS1) to inhibit mTORC1 [42].

In this study, we set out to differentiate whether it is AMPK or AXIN lysosomal translocation triggered by FBP-unoccupied aldolase that plays a dominant role in switching off mTORC1 under glucose starvation. This also pertains to the issue of the autonomy of glucose availability itself in the regulation of mTORC1, and of AMPK.

## Results

### Glucose availability itself controls the lysosomal translocation of AXIN

**Figure 1:**
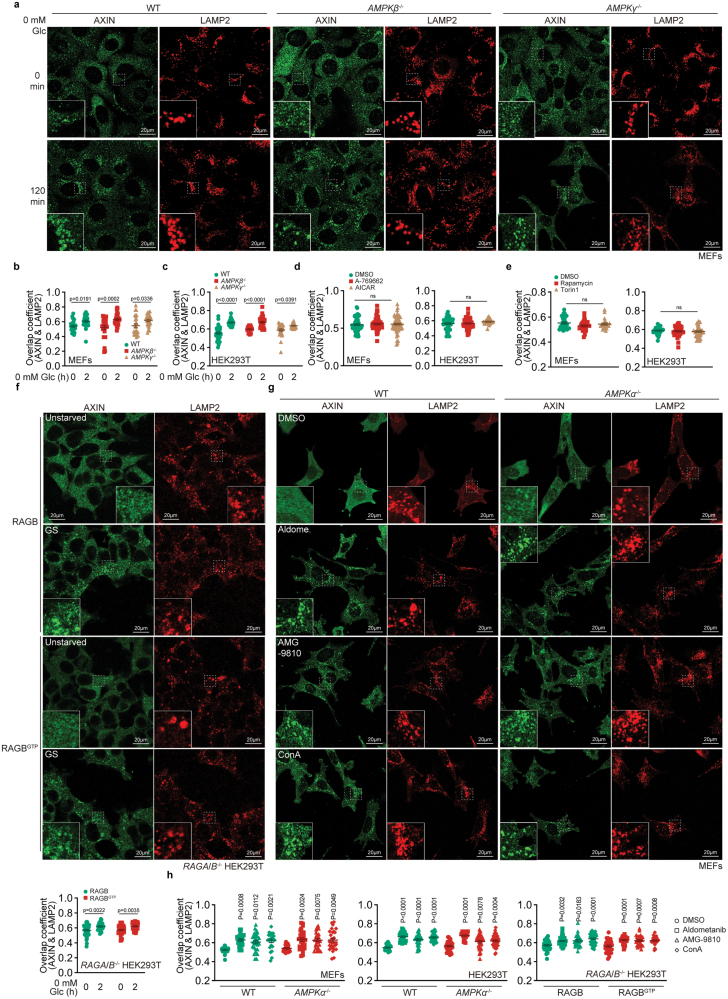
Glucose/FBP availability controls lysosomal translocation of AXIN. (a–c) Knockout of AMPK does not prevent the lysosomal translocation of AXIN under glucose starvation conditions. MEFs (a, b) and HEK293T cells (c) with *AMPKβ1*/*2* and *AMPKγ1*/*2*/*3* knocked out, along with their wild-type controls, were starved for glucose (Glc) for 2 h. AXIN and the lysosomal marker lysosome-associated membrane protein 2 (LAMP2) were stained, and images were taken by confocal microscopy. Representative images of MEFs are shown in (a), and the areas defined by dashed boxes on each representative image are enlarged as insets. See also representative images of HEK293T cells in Supplementary Fig. S1b. The degrees of co-localization between AXIN and LAMP2 were quantified by the Mander’s overlap coefficients, which are plotted as means ± SEM in (b, c), *n* = 25−34 (b) or 25–35 (c) cells, with *P* values calculated by two-way ANOVA, followed by Sidak. (d, e) Forced activation of AMPK or inhibition of mTORC1 in high glucose conditions does not trigger AXIN translocation. MEFs (left) and HEK293T cells (right) were treated with 200 μmol/L A-769662 (d), 0.6 mmol/L AICAR (d), 100 nmol/L rapamycin (e), 250 nmol/L Torin1 (e), or DMSO control for 2 h, followed by determination of the co-localization between AXIN and LAMP2 as in (a). Mander’soverlap coefficients are plotted as means ± SEM, *n* = 53−60 (d, MEFs), 26–53 (d, HEK293T cells), 24–29 (e, MEFs) cells, or 31–38 (e, HEK293T cells), with *P* values calculated by one-way ANOVA, followed by Tukey (d), Sidak (e, HEK293T cells), or Dunn’s (e, MEFs); ns, not significant. See also representative images in Supplementary Figs. S1d and S2b. (f) Forced activation of mTORC1 under glucose starvation conditions does not prevent AXIN translocation. HEK293T cells with knockout of both *RAGA* and *RAGB* were re-introduced with RAGB^GTP^ or wild-type RAGB as a control, followed by starved for glucose (GS) for 2 h. Co-localization between AXIN and LAMP2 was then determined, and the Mander’s overlap coefficients are plotted as means ± SEM, *n* = 30−47 cells, with *P* values calculated by two-way ANOVA, followed by Tukey. (g, h) Inhibition of aldolase-TRPV-v-ATPase-Ragulator-RAG axis mimics glucose starvation to trigger AXIN translocation. MEFs with *AMPKα1*/*2* knocked out (g, h) HEK293T cells with *AMPKα1*/*2* knocked out (h), or HEK293T cells with *RAGA*/*B* knocked out and then RAGB^GTP^ re-introduced (h), were treated with 10 nmol/L Aldometanib (Aldome.), 5 μmol/L AMG-9810, 5 μmol/L conA, or DMSO control for 2 h, followed by determination of the co-localization between AXIN and LAMP2. Representative images of MEFs are shown in (g), and HEK293T in Supplementary Fig. S3. In (h), Mander’s overlap coefficients are plotted as means ± SEM, *n* = 22−28 (MEFs), 20–43 (HEK293T cells), or 24–50 (*RAGA*/*B*^−/−^ HEK293T cells) cells, with *P* values calculated by two-way ANOVA, followed by Tukey, except those *RAGA*/*B*^−/−^ HEK293T cells Sidak. Experiments in this figure were performed three times independently.

We first determined the relationship among AMPK, mTORC1, and the availability of glucose-FBP in controlling the lysosomal translocation of AXIN. As previously shown, AMPKα, the catalytic subunit of AMPK [43], is not involved in the translocation of AXIN under glucose starvation conditions [19]. Here, we determined whether the regulatory/scaffolding subunits of AMPK [44–46], AMPKβ and AMPKγ [43, 47], are required for AXIN translocation by knocking out *AMPKβ* (both *AMPKβ1* and *AMPKβ2*) and *AMPKγ* (*AMPKγ1*, *AMPKγ2*, and *AMPKγ3*) together in mouse embryonic fibroblasts (MEFs; see Supplementary Fig. S1a for validation) and HEK293T cells (validated in [48]), and found that all the regulatory subunits of AMPK were not required for AXIN translocation under glucose starvation (Fig. 1a–c and Supplementary Fig. S1b), indicating that AXIN translocation does not rely on a prior interaction with the lysosomally localized AMPK complex. We also found that forced activation of AMPK, as assessed by the levels of phosphorylated AMPKα (p-AMPKα) and its substrate ACC (p-ACC) [49] (Supplementary Fig. S1c), with a moderate dose of the AMP mimetic AICAR [48, 50] or with another allosteric activator, A-769662 [51], failed to trigger the lysosomal translocation of AXIN in MEFs and HEK293T cells (Fig. 1d and Supplementary Fig. S1d). Similarly, prior inhibition of mTORC1 by rapamycin [52] or Torin1 [53], assessed by the levels of the phosphorylated mTORC1 substrate, S6K (p-S6K) [54] (Supplementary Fig. S2a), failed to trigger AXIN translocation in MEFs and HEK293T cells (Fig. 1e, Supplementary Fig. S2b). In addition, re-introduction of constitutive active RAGB^GTP^ [26, 55] in RAG-deficient HEK293T cells (double knockout of both *RAGA* and *RAGB* in HEK293T cells, leaving these cells with no RAG activity [17, 26]; see validation data in Supplementary Fig. S2c) rendered mTORC1 insensitive to inhibition by glucose starvation (Supplementary Fig. S2d), and did not block the lysosomal translocation of AXIN (Fig. 1f). Therefore, the activation of AMPK and the inhibition of mTORC1 under glucose starvation conditions do not play a causal role in AXIN translocation.

In contrast, the lysosomal glucose sensing pathway plays a striking role in the lysosomal translocation of AXIN, as prevention of FBP binding to aldolase by treatment of the cells with aldometanib [56], inhibition of TRPV by AMG-9810 [57, 58], or inhibition of v-ATPase by concanamycin A (conA [59, 60]) all mimicked the effects of glucose starvation and induced the lysosomal translocation of AXIN (Fig. 1g and h). Effects of aldometanib, AMG-9810 and conA on AXIN translocation were also observed in *AMPKα*^−/−^ and RAGB^GTP^-expressing cells (Fig. 1g and h and Supplementary Fig. S3). Therefore, it is the lack of glucose availability itself that plays an autonomous role in triggering AXIN translocation.

### AXIN plays a dominant role in mTORC1 inhibition

**Figure 2:**
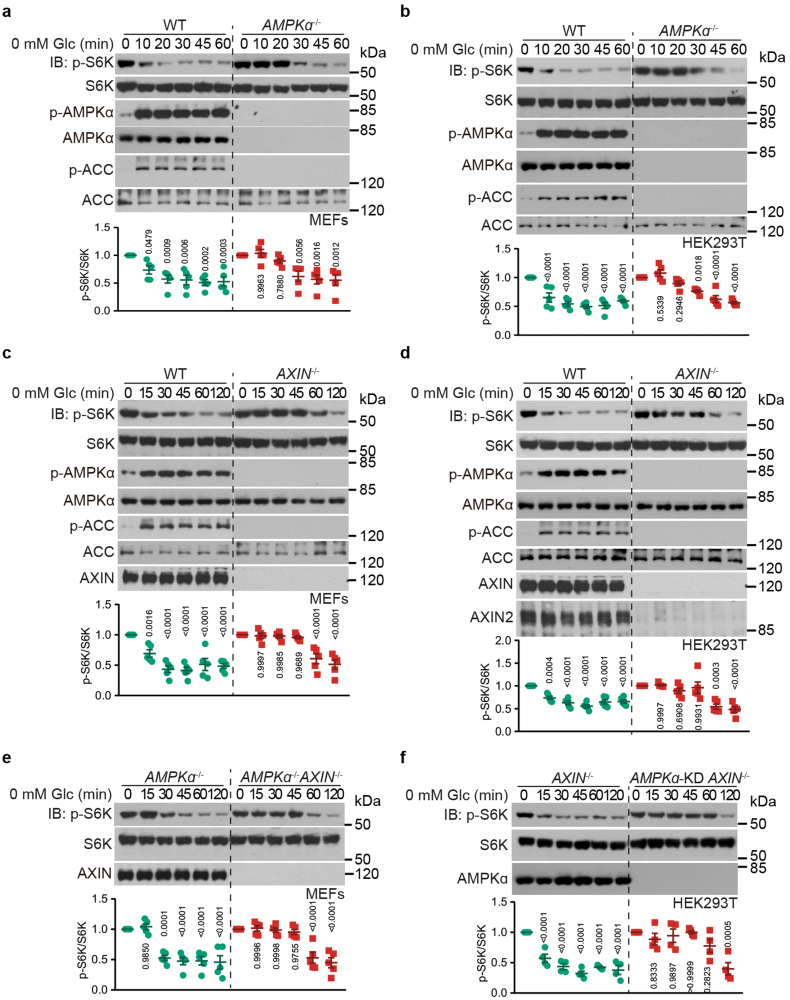
AXIN overshadows AMPK in mTORC1 inhibition. MEFs (a, c, e) or HEK293T cells (b, d, f) with *AMPKα1*/*2* knockout (a, b), *AXIN* knockout (c, d), knockout of both *AMPKα1*/*2* and *AXIN* (e), or knockdown (KD) of *AMPKα1*/*2* and knockout of *AXIN* (f) were starved for glucose for the indicated time periods. Cells were then lyzed, and the mTORC1 activity was assessed by determining the p-S6K levels by immunoblotting, followed by densitometry analysis [data are means ± SEM, *n* = 4 (f) or 5 (others), with *P* values calculated by one-way ANOVA, followed by Dunnett, except that *AMPKα*^−/−^ in (a) and *AXIN*^−/−^ in (d) by Dunn’s, compared the ratios of p-S6K:S6K between unstarved group and glucose-starved group at each time point]. Experiments in this figure were performed three times independently.

We next compared the respective importance of AXIN and AMPK in the inhibition of mTORC1 by evaluating the dynamics of mTORC1 inhibition under low glucose conditions. We found that the half-life of mTORC1, as determined by the time duration of glucose starvation required for the decline of p-S6K levels to reach half of their maximal levels (mid-point of p-S6K), was around 15 min in MEFs and HEK293T cells, which is around the time when AMPK was activated (Fig. 2a and b). In the absence of AMPK activity due to knockout of both *AMPKα1* and *AMPKα2*, the inhibition of mTORC1 could still be observed under glucose starvation, albeit at a slower rate compared with wild-type cells, with the mid-point of p-S6K extended to approximately 30 min (Fig. 2a and b). In comparison, depletion of AXIN1 (also known as AXIN) in MEFs (expressing only AXIN1, as validated in [48]), or AXIN2 (also known as *Conductin* or *AXIL*) in *AXIN1*^−/−^ HEK293T cells (with redundant expression and function of both AXIN1 and AXIN2, see [48, 61, 62]) leads to a further slowed velocity of mTORC1 inhibition with the mid-point of p-S6K being extended to approximately 60 min (Fig. 2c and d). These data indicate that AXIN exerts a more pronounced role in mTORC1 inhibition compared to AMPK. Consistent with these results, knockout of *AXIN* in *AMPKα*^−/−^ MEFs, or knockdown of *AMPKα* in *AXIN1*/*2*^−/−^ HEK293T cells extended the mid-point of p-S6K to 1 h from 30 min in their respective parental cells under glucose starvation, which is similar to the effects seen with AXIN deficiency alone (Fig. 2e and f). These results further validate the notion that AXIN plays a more prominent role than AMPK in mTORC1 inhibition during glucose starvation.

### AXIN, but not AMPK, facilitates the lysosomal dissociation of mTORC1 under glucose starvation conditions

**Figure 3:**
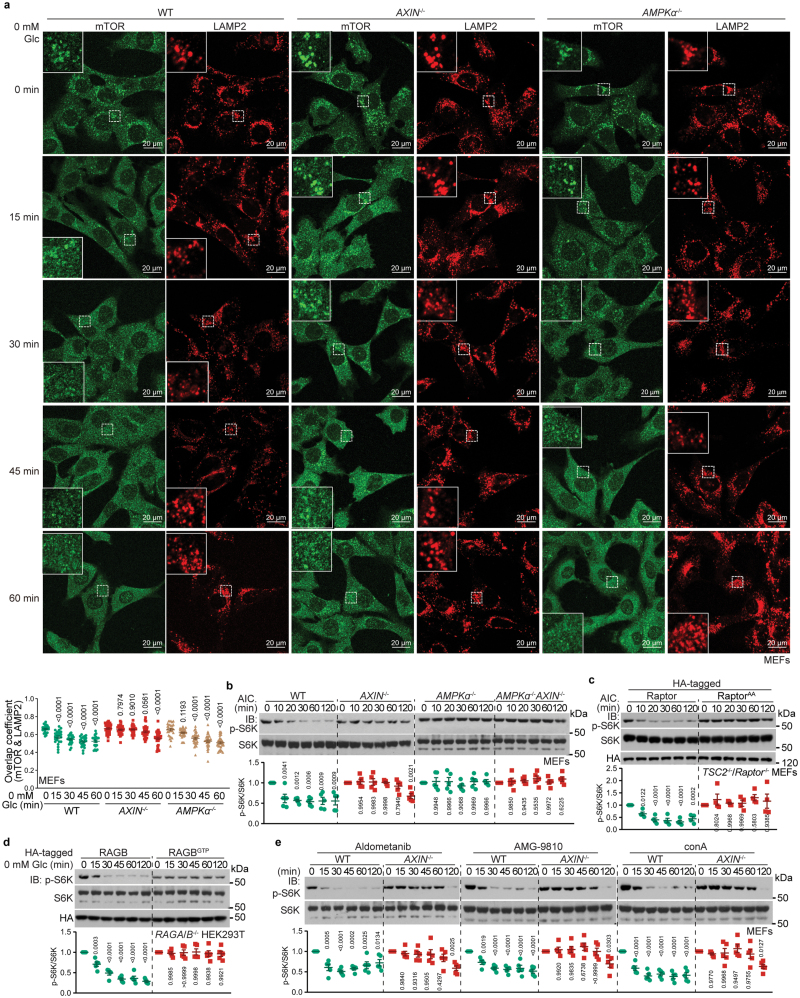
AXIN lysosomal translocation dissociates mTORC1 from lysosome. (a) AXIN, but not AMPK, facilitates mTORC1 dissociation from the lysosome under glucose starvation conditions. MEFs with knockout of *AXIN* or *AMPKα1*/*2* were starved for glucose for the indicated time periods, followed by determination of the co-localization between mTOR and LAMP2 by confocal microscopy. Mander’s overlap coefficients are plotted as means ± SEM, *n* = 21−31 cells, with *P* values calculated by one-way ANOVA, followed by Dunnett. (b, c) AICAR, unable to dissociate mTORC1 from the lysosome, inhibits mTORC1 through AMPK. MEFs with knockout of *AXIN* (b), knockout of *AMPKα1*/*2* (b), knockout of both *AMPKα1*/*2* and *AXIN* (b), or knockout of both *TSC2* and *Raptor* with re-introduced Raptor^AA^ (c) were treated with 0.6 mmol/L AICAR (AIC.) for the indicated time periods, followed by determining the p-S6K and S6K levels by immunoblotting [data are quantified and are shown as means ± SEM, *n* = 4 (c) or 5 (others), with *P* values calculated by one-way ANOVA, followed by Dunnett, compared p-S6K:S6K between DMSO group and AICAR group at each time point]. (d) Forced lysosomal anchoring of mTORC1 is unable to be inhibited by glucose starvation. HEK293T cells with *RAGA* and *RAGB* knocked out were re-introduced with RAGB^GTP^, or wild-type RAGB as a control, followed by starvation for glucose for the indicated time periods. p-S6K and S6K levels were then determined by immunoblotting (data are quantified and are shown as means ± SEM, *n* = 5, with *P* values calculated by one-way ANOVA, followed by Dunnett, compared as in Fig. 2). (e) AXIN facilitates mTORC1 inhibition via the aldolase-TRPV-v-ATPase- Ragulator-RAG axis. MEFs with *AXIN* knocked out were treated with 10 mmol/L Aldometanib, 5 μmol/L AMG-9810, 5 μmol/L conA or DMSO as control for 2 h, followed by determination of the p-S6K and S6K levels (data were quantified and are shown as means ± SEM, *n* = 5, with *P* values calculated by one-way ANOVA, followed by Dunnett, compared as in b). Experiments in this figure were performed three times independently.

We have previously shown that AXIN translocates to the lysosomal surface to activate AMPK and to concomitantly facilitate the dissociation of mTORC1 from RAGs [19]. Here, we set out to determine whether AMPK activation exerts a role in mTORC1 dissociation. We found that *AMPKα* knockout had no effect on the lysosomal dissociation of mTORC1 under glucose starvation, while *AXIN* knockout strongly retarded the dissociation (Fig. 3a). Therefore, AMPK is only involved in promoting the inhibition of Rheb by TSC2 while directly phosphorylating Raptor to abolish mTORC1 activity, rather than altering its localization to the lysosome.

We also used AICAR at a concentration that mimics a mild increase of AMP levels (moderate dose) to allow for the activation of the cytosolic pools of AMPK in addition to the lysosomal pools [48, 50]. Unlike glucose starvation, moderate-dose AICAR treatment did not cause a lysosomal translocation of AXIN (Fig. 1d and Supplementary Fig. S1d). Instead, the effects of AICAR depend on AXIN to tether LKB1 to induce the phosphorylation of both the cytosolic and lysosomal pools of AMPK [21, 48]. AICAR also failed to dissociate mTORC1 from the lysosome (Supplementary Fig. S4a and b). Without AXIN translocation to and mTORC1 dissociation from the lysosome, it is likely that AICAR inhibits mTORC1 via the kinase activity of AMPK, as knockout of *AMPKα* almost completely blocked the AICAR-induced inhibition of mTORC1, with the half-life of p-S6K extending to more than 120 min (Fig. 3b).

Consistent with these results, in cells with knockout of *AXIN*, a moderate dose of AICAR still depends on AXIN for AMPK activation as AICAR failed to activate AMPK in the knockout cells [48] or to facilitate mTORC1 inhibition (Fig. 3b). In line with the kinase activity of AMPK being responsible for the phosphorylation of TSC2 and Raptor, re-introduction into *Raptor*/*TSC2*^−/−^ MEFs of Raptor^AA^, a mutant that is incapable of being phosphorylated by AMPK [38] (validated in Supplementary Fig. S4c), in the presence of AICAR, extended the half-life of p-S6K to an extent similar to that of *AMPKα* knockout (Fig. 3b and c). These data also indicate that under glucose starvation conditions, the pronounced role of AXIN over AMPK in depleting mTORC1 activity is exerted through the dissociation of mTORC1 from the lysosome. Furthermore, re-introduction of RAGB^GTP^ into *RAGA/B*^−/−^ HEK293T cells blocked the inhibition of mTORC1 (assessed by p-S6K) under glucose starvation conditions (Fig. 3d), validating the notion that lysosomal dissociation of mTORC1 promoted by AXIN plays a primary role in the inhibition of mTORC1 under glucose starvation conditions.

Compared with knockout of *AMPK* and *AXIN* (Fig. 2a–d), RAGB^GTP^ expression was more effective in retarding glucose starvation-induced mTORC1 inhibition (Fig. 3d), which may be achieved through maintaining Rheb in an active state; for ­example, through facilitating the dissociation of TSC2 from Rheb [63]. As RAGB^GTP^ did not prevent AXIN translocation (­Fig. 1f), but blocked AXIN-mediated mTORC1 dissociation and inhibition (Fig. 3d and [19, 31]), it can be concluded that the inhibition of RAGs is a consequence (downstream event) of AXIN docking onto the lysosomal v-ATPase-Ragulator complex; that is, as a result of facilitating the inhibition of Ragulator activity to accelerate the release of GTP from RAGC [19]. Supporting this notion, aldometanib, AMG-9810, and conA, which all cause AXIN lysosomal translocation by mimicking the effects of low glucose-FBP level on the aldolase-TRPV-v-ATPase-Ragulator-RAG axis, all showed poor inhibition of mTORC1 in *AXIN* knockout cells (Fig. 3e).

### AMPK inhibits the residual, Rheb-dependent mTORC1 activity in RAGs-null cells

**Figure 4:**
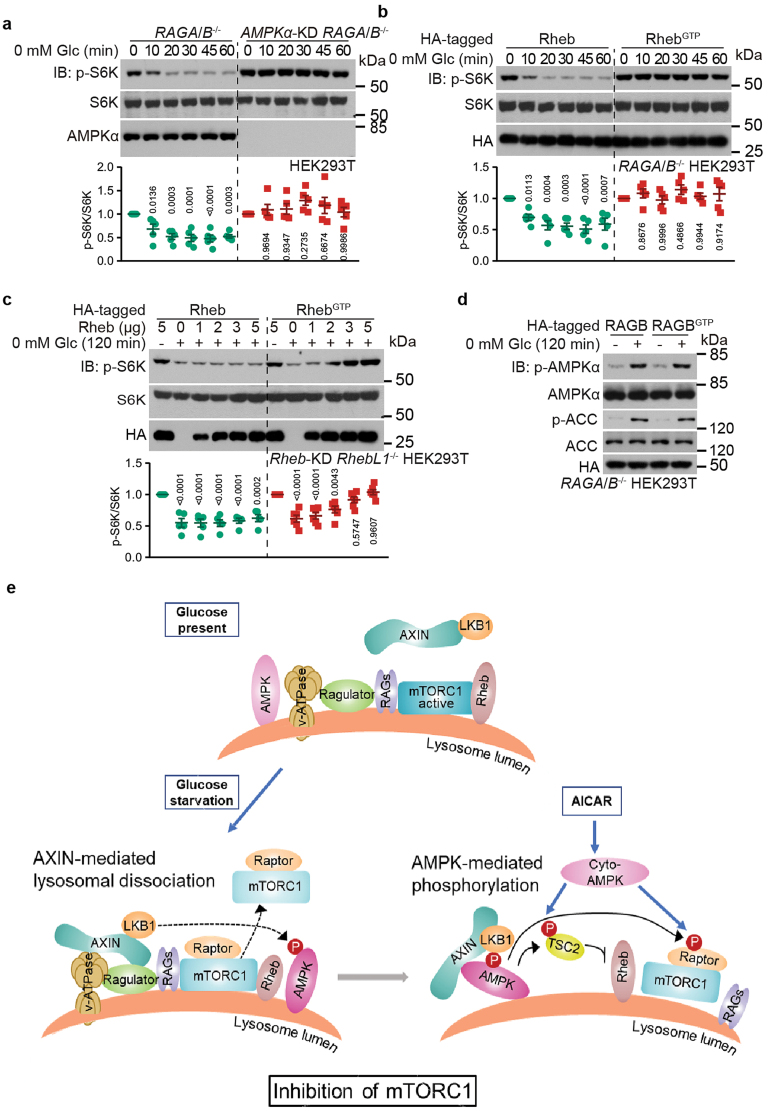
AMPK inhibits the residual mTORC1 activity in RAGs-null cells. (a) AMPK is required for mTORC1 inhibition in RAGs-null cells under glucose starvation conditions. HEK293T cells and *AMPKα1*/*2*-knockdown HEK293T cells, both with knockout of *RAGA*/*B* were starved for glucose for the indicated time periods, followed by determination of the p-S6K and S6K levels (data were quantified and are shown as means ± SEM, *n* = 5, with *P* values calculated by one-way ANOVA, followed by Dunnett, compared as in Fig. 2). (b) Inhibition of mTORC1 in RAGs-null cells under glucose starvation conditions is a consequence of Rheb inhibition. *RAGA*/*B*^−/−^ HEK293T cells with Rheb^GTP^ stably expressed were starved for glucose for the indicated time periods, followed by determination of the p-S6K and S6K levels (data were quantified and are shown as means ± SEM, *n* = 5, with *P* values calculated by one-way ANOVA, followed by Dunnett, compared as in Fig. 2). (c) Rheb^GTP^ attenuated glucose starvation-mediated mTORC1 inhibition. *RhebL1*^−/−^ HEK293T cells with *Rheb* knocked down were transfected with Rheb^GTP^ at desired amounts, followed by determination of the p-S6K and S6K levels (data were quantified and are shown as means ± SEM, *n* = 5, with *P* values calculated by two-way ANOVA, followed by Tukey, compared as in Fig. 2). (d) Forced activation of mTORC1 under glucose starvation condition does not prevent AMPK activation. *RAGA*/*B*^−/−^ HEK293T cells with RAGB^GTP^ re-introduced were starved for glucose for 2 h. AMPK activity was then assessed by immunoblotting for the p-AMPK and p-ACC levels. (e) Schematic diagram showing the roles of AXIN and AMPK in mTORC1 inhibition under glucose starvation conditions. In high glucose conditions, mTORC1 is in an active state by interacting with both RAGs and the allosteric activator Rheb (upper). Under glucose starvation hence low FBP conditions, AXIN forms complex with the v-ATPase-Ragulator complex on the surface of the lysosome, which is primed by the FBP-unoccupied aldolase. AXIN then facilitates the inhibition of RAGs, thereby promoting the dissociation of mTORC1 from the lysosome (lower left). AXIN also brings along LKB1 to the vicinity of lysosome-localized AMPK and activates it. Through phosphorylating TSC2 and Raptor, AMPK further inhibits the activity of mTORC1 (lower right). Note that AICAR, which activates both the lysosomal and the cytosolic (cyto) pools of AMPK, strongly inhibits mTORC1, regardless of its forced lysosomal localization by RAGB^GTP^ (lower right). Experiments in this figure were performed three times independently.

We found that in *RAGA*/*B*^−/−^ HEK293T cells, the p-S6K signal could still be detected and the half-life of p-S6K is similar to the wild-type HEK293T cells under glucose starvation conditions (Fig. 4a). Knockdown of *AMPKα* almost completely blocked the inhibition of mTORC1 in *RAGA*/*B*^−/−^ HEK293T cells (Fig. 4a). In these *RAGA*/*B*^−/−^ HEK293T cells, Rhebs are the remaining anchor and activator for mTORC1, as the expression of the GTP-locked mutant Rheb-S16H, or Rheb^GTP^ [64], attenuated glucose starvation-mediated mTORC1 inhibition (Fig. 4b). It should be noted that unlike RAGs, which are exclusively localized on the lysosome [17], Rhebs may persist on other endomembrane compartments, such as the ER [65–68], the Golgi apparatus [65–67, 69–71], peroxisomes [72], and mitochondria [73, 74]. Possibly due to the multiple localizations [65, 67, 68, 71], Rheb^GTP^ expression only slightly increased the lysosomal translocation of mTORC1 (Supplementary Fig. S4d). In addition, when we re-introduced the constitutively active Rheb^GTP^ into Rheb-deficient HEK293T cells (generated by knocking down *Rheb* in *RhebL1*^−/−^ HEK293T cells and thus depleting the activity of Rheb [75]; validated in Supplementary Fig. S4c), Rheb^GTP^ at high levels attenuated glucose starvation-mediated mTORC1 inhibition (Fig. 4c). In contrast, in wild-type HEK293T cells, it is RAGs that dominantly regulate mTORC1 as RAGB^GTP^ completely blocked glucose starvation-induced mTORC1 inhibition (Fig. 3d). Therefore, Rhebs and RAGs may stoichiometrically regulate mTORC1. In particular, when expressed at a level higher than RAGs, Rhebs play a dominant role in anchoring and maintaining the activity of mTORC1, such that in glucose starvation states, AMPK would be needed for the inhibition of mTORC1 through TSC2 phosphorylation.

### Forced activation of mTORC1 does not change AMPK activity

We also evaluated whether forced activation of mTORC1 has any effect on AMPK activation in glucose starvation conditions. When we re-introduced RAGB^GTP^ in *RAGA*/*B*^−/−^ HEK293T cells, we found that there was full activation of AMPK under glucose starvation conditions (Fig. 4d). Together with the results showing an intact AXIN translocation in *RAGA*/*B*^−/−^ HEK293T cells expressing RAGB^GTP^ under glucose starvation conditions (Fig. 1f), we conclude that there is not a reverse signaling in which mTORC1 inhibits the activation of AMPK.

**Discussion**

In this study, we have shown that the lysosomal translocation of AXIN plays a dominant role in the inhibition of mTORC1 under glucose starvation conditions. As shown previously, AXIN can form interactions with various subunits of v-ATPase and with the pentameric Ragulator complex in response to glucose starvation [19]. Modulating the lysosomal glucose sensing pathway to mimic the glucose starvation or low FBP level state, such as inhibiting v-ATPase and TRPV, readily causes AXIN translocation. We have further shown that AMPK does not play a role in AXIN translocation, as knockout of AMPK or its forced activation by AICAR or A-769662 in high glucose conditions fails to cause the lysosomal translocation of AXIN. Similarly, the activity of mTORC1 has no role in AXIN lysosomal translocation, as forced activation of mTORC1 by expression of RAGB^GTP^ did not abrogate the lysosomal translocation of AXIN under glucose starvation condtions, nor did inhibition of mTORC1 by rapamycin or Torin1 (an ATP-competitive mTOR inhibitor of the quinoline class, which inhibits phosphorylation of both mTORC1 and mTORC2) in high glucose.

We further dissected how AXIN translocation induces mTORC1 inhibition and found that it is responsible for dissociating mTORC1 from the lysosomal surfaces through binding to Ragulator to inhibit RAGs, in addition to mediating AMPK activation (Fig. 4e). This explains the pronounced effect of the *AXIN* knockout over the *AMPKα* knockout on retardation of mTORC1 inhibition under glucose starvation conditions. Therefore, the hierarchical inhibition of mTORC1 under glucose starvation conditions is mediated primarily by AXIN translocation to the lysosome to dissociate mTORC1 with a secondary mechanism involving AMPK phosphorylation of TSC2 and Raptor. The secondary mechanism is particularly true when intracellular AMP is elevated, as seen with administration of AICAR, during which no AXIN translocation and hence mTORC1 dissociation occurs, and thus the role of AXIN is subsumed by the activity of AMPK for mTORC1 inhibition.

It is interesting to observe that AICAR overrides the roles of AXIN in mTORC1 dissociation and inhibition; that is, it inhibits mTORC1 without the need to dissociate it from the lysosome. This can also be seen from the observations by others that even when mTORC1 is stably anchored onto the lysosome, for example, by expressing constitutively active RAGs, it can be inhibited by AICAR [31], which resembles moderately severe stress as opposed to glucose starvation [48]. Such an effect might be attributed to the larger scale activation of AMPK caused by AICAR than that of glucose starvation [48]. As a result, the importance of AMPK (stoichiometrically) overwhelms AXIN in mTORC1 inhibition, rendering it as a bypass mechanism for the effects of RAGs on mTORC1 regulation, which is similar to the dominant effects of Rheb that is observed when it is expressed at high levels (Fig. 4a–c). In summary, our data support the notion that AXIN plays a major role in mTORC1 inhibition, primarily through translocation, to elicit a combined effect of AMPK activation and RAG inhibition.

We have also shown that mTORC1, when maintained constitutively active under glucose starvation conditions through expression of RAGB^GTP^, does not, in turn, inhibit the activation of AMPK. However, it is noteworthy that mTORC1 has been reported to inhibit the basal activity of AMPK in high glucose conditions, which is likely mediated by Ca^2+^/calmodulin-dependent protein kinase kinase 2 (CaMKK2), an alternative upstream kinase of AMPK that is responsible for Ca^2+^-induced AMPK activation [76–78]. It has been shown that during refeeding, leptin-induced mTORC1 activation inhibits AMPK via S6K-mediated phosphorylation and inhibition of AMPKα2 in the hypothalamus [79]. It has also been shown that amino acid withdrawal, which leads to mTORC1 inhibition (see below for details), can stimulate AMPK activity, particularly AMPKα2-based activity [80, 81]. Very recently, it was reported that in islet β-cells from chronic hyperglycemic mice, there is an accumulation of FBP and DHAP, resulting in a constitutively high activity of mTORC1 and S6K-dependent inhibition of AMPK [12].

The observations that mTORC1 inhibition can still occur in AXIN-deficient cells under glucose starvation, albeit very slowly, suggest that AXIN acts to facilitate the inhibition of RAGs under glucose starvation conditions, consistent with our earlier report that Ragulator shows basal inhibitory activity towards RAGB [19]. It has now become evident that the inhibitory activity of Ragulator towards RAGA/B is actually achieved through promoting the release of GTP, rather than the release of GDP, from RAGA/B, and Ragulator has therefore been re-defined as a “non-canonical guanine exchange factor (GEF)” of RAGs [24]. The basal non-canonical GEF activity of Ragulator may possibly be attributed to FBP-unoccupied aldolase that influences the conformation of v-ATPase [15, 20]. In addition, other molecular changes that result from glucose withdrawal may mediate the mTORC1 inhibition that occurs in the absence of AXIN. It has also been shown that under glucose starvation conditions, the glycolytic enzyme glyceraldehyde-3-phosphate dehydrogenase (GAPDH) binds to and inhibits Rheb when unoccupied with its substrate G3P [82]. It has also been shown that phosphofructokinase 2 loses its affinity towards mTORC1 when unoccupied with fructose-2,6-bisphosphate (F-2,6-BP), which prevents mTORC1 from binding to RAGs [83]. These intricate mechanisms together assure the fine-tuned inhibition of mTORC1 under glucose starvation conditions.

Apart from glucose, amino acids can also activate mTORC1 through the v-ATPase-Ragulator-RAGs axis. However, different from glucose-FBP availability that is transmitted solely through the aldolase-TRPV complex, the upstream factors for sensing and signaling amino acid availability to v-ATPase are numerous. For example, alanine, proline, and glycine use the lysosomal SLC36A1/proton-assisted amino acid transporter (PAT1) transporters, while arginine uses SLC38A9 (reviewed in [84]). In addition, some amino acids can be sensed by sensors that directly regulate RAGs, rather than v-ATPase, such as leucine (at high concentrations) by Sestrin and the leucyl-tRNA synthetase (LRS), and methionine by *S*-adenosylmethionine sensor upstream of mTORC1 (SAMTOR) (reviewed in [85]). Low concentrations of leucine can be sensed by secretion associated Ras related GTPase 1 homolog B (SAR1B), which also signals to RAGs [86]. Moreover, studies have also suggested that the TSC2-Rheb axis, in addition to the v-ATPase-Ragulator-RAGs axis, can also be regulated by amino acids [63], such as arginine [87]. Furthermore, some amino acids, such as glutamine, can regulate mTORC1 in a v-ATPase-dependent, Ragulator- and RAG-independent manner [88]. Furthermore, amino acid availability does not regulate the activity of TRPV [15]. In addition, regulation of mTORC1 by amino acids does not require AXIN [19], and amino acid starvation does not promote the lysosomal translocation of AXIN (Supplementary Fig. S5). Finally, amino acids have been shown to also regulate AMPK, although none of the mechanisms reported to date (at least in mammals) are related to the v-ATPase-Ragulator axis. In addition to the inhibition of AMPK mediated by mTORC1/S6K as discussed above [80, 81], it has been shown that high cysteine can be sensed by cysteinyl-tRNA synthetase to inhibit CaMKK2, thereby inhibiting AMPK [45]. It has also been reported that high concentrations of amino acids, particularly glutamine and alanine, can elevate AMP for activating AMPK after entering the urea cycle [89, 90]. High leucine, similarly, also activates AMPK in an AMP-dependent manner (summarized in [91]). In addition, acute re-addition of amino acids can activate AMPK in Ca^2+^- and CaMKK2-dependent manners [92].

## Materials and methods

### Antibodies

Rabbit anti-phospho-p70 S6K-T389 [cat. #9234, 1:1000 for immunoblotting (IB)], anti-p70 S6K (cat. #2708, 1:1000 for IB), anti-phospho-AMPKα-T172 (cat. #2535, 1:1000 for IB), anti-AMPKα (cat. #2532, 1:1000 for IB), anti-phospho-ACC-Ser79 (cat. #3661, 1:1000 for IB), anti-ACC (cat. #3662, 1:1000 for IB), anti-AMPKβ (cat. #4150, 1:1000 for IB), anti-AXIN1 (cat. #2074, 1:1000 for IB), anti-AXIN2 (cat. #2151, 1:1000 for IB), anti-RAGA (cat. #4357, 1:1000 for IB), anti-RAGB (cat. #8150, 1:1000 for IB), anti-Rheb (cat. #13879, 1:1000 for IB), anti-Raptor (cat. #2280, 1:1000 for IB), anti-TSC2 (cat. #4308, 1:1000 for IB), and anti-mTOR [cat. #2983, 1:100 for immunofluorescent staining (IF)] antibodies were purchased from Cell Signaling Technology. Rabbit anti-AMPKγ1 (cat. ab32508, 1:1,000 for IB), rat anti-lysosome-associated membrane protein 2 (LAMP2) (for MEFs; cat. ab13524, 1:120 for IF), and mouse anti-LAMP2 (for HEK293T cells; cat. ab25631, 1:120 for IF) antibodies were purchased from Abcam. Rabbit anti-AMPKγ2 (cat. NBP1-89324, 1:1000 for IB) was purchased from Novus Biologicals. Rabbit anti-AMPKγ3 (cat. AP13603PU-N, 1:500 for IB) was purchased from Origene. Rabbit anti-RhebL1 (cat. SAB2102001, 1:1000 for IB) antibody was purchased from Sigma. Mouse anti-tubulin (cat. #66031-1-Ig, 1:20,000 for IB) antibody was purchased from Proteintech. Goat anti-AXIN (cat. sc-8567, 1:120 for IF) and mouse anti-HA (cat. sc-7392, 1:1000 for IB) antibodies were purchased from Santa Cruz Biotechnology. The HRP-conjugated goat anti-mouse IgG (cat. 115-035-003, 1:5000 for IB) and goat anti-rabbit IgG (cat. 111-035-003, 1:5000 for IB) antibodies were purchased from Jackson ImmunoResearch. Alexa Fluor 488 donkey anti-goat IgG (cat. A11055, 1:100 for IF), Alexa Fluor 594 donkey anti-rat IgG (cat. A21209, 1:100 for IF), and Alexa Fluor 488 goat anti-rabbit IgG (cat. A11008, 1: 100 for IF) antibodies were purchased from Thermo.

### Chemicals

DMSO (cat. D2650), glucose (cat. G7021), CsCl (cat. 289329), NaHCO_3_ (cat. S5761), Trizma^®^ base (Tris; cat. T1503), NaCl (cat. S7653), EDTA (cat. E6758), EGTA (cat. E3889), SDS (cat. 436143), formaldehyde solution (formalin; cat. F8775), sodium pyrophosphate (cat. P8135), β-glycerophosphate (cat. 50020), AICAR (cat. A9978), A-769662 (cat. SML2578), AMG-9810 (cat. A2731), formaldehyde solution (formalin; cat. F8775), phosphate-buffered saline (PBS; cat. P5493), Triton^TM^ X-100 (cat. T9284), Tween-20 (cat. P9416), polybrene (cat. H9268), BSA (cat. A2153), and Non-Fat-Dried Milk bovine (cat. M7409) were purchased from Sigma. Polyethylenimine (PEI; cat. 23966) was purchased from Polysciences. Rapamycin (cat. S1039) and Torin1 (cat. S2827) were purchased from Selleck. Aldometanib was synthesized as described previously [56], and now available at MedChemExpress (cat. HY-148189), GLPBIO (cat. GC66024), and CymitQuimica (cat. TM-T60122). Concanamycin A (conA, cat. 11050) was purchased from Cayman. WesternBright^TM^ ECL and Peroxide solutions (cat. 210414-73) were purchased from Advansta. Protease inhibitor cocktail (cat. 70221) was purchased from Roche. ProLong™ Diamond Antifade Mountant (cat. P36970), Lipofectamine^TM^ 2000 (cat. 11668500), DMEM, high glucose (cat. 12800082), DMEM, no glucose (cat. 11966025), MEM non-essential amino acids solution (cat. 11140050), fetal bovine serum (cat. 10099141C), penicillin-streptomycin (cat. 15140163), sodium pyruvate (cat. 11360070), ProLong Diamond Antifade Mountant (cat. P36970), and Prestained Protein MW Marker (cat. 26612) were purchased from Thermo. Normal goat serum (NGS; cat. SL038) was purchased from Solarbio. Amino acid-free DMEM was customized from Shanghai BasalMedia Technologies Co.,LTD.

The concentrations of agonists and inhibitors for AMPK and mTORC1 are as follows: 200 μmol/L A-769662, 0.6 mmol/L AICAR, 100 nmol/L Rapamycin, 250 nmol/L Torin 1, 10 nmol/L Aldometanib, 5 μmol/L AMG-9810, and 5 μmol/L Concanamycin A. DMSO was used as a control for all experiments involving the use of the individual chemicals.

### Plasmids

Full-length cDNAs used in this study were obtained either by PCR using cDNA from MEFs, or by purchasing from Origene or Sino Biological. Mutations of RAGB, Raptor, and Rheb were performed by PCR-based site-directed mutagenesis using PrimeSTAR HS polymerase (cat. R40A, Takara). Expression plasmids for various epitope-tagged proteins were constructed in the pcDNA3.3 vector for transfection (ectopical expression in mammalian cells) or in the pBOBI vector for lentivirus packaging (stable expression in mammalian cells). PCR products were verified by sequencing (Invitrogen, China). The lentivirus-based vector pLV-H1-EF1a-puro was used for expression of siRNA in MEFs and HEK293T cells. The sequence for siRNA of human *Rheb* was: 5ʹ-CCAGGTTGGATTCCAGAAA-3ʹ. All plasmids used in this study were purified by CsCl density gradient ultracentrifugation method.

### Cell lines

In this study, no cell line used is on the list of known misidentified cell lines maintained by the International Cell Line Authentication Committee (iclac.org/databases/cross-contaminations). MEFs were established by introducing SV40 T antigen via lentivirus into cultured primary embryonic cells from male mouse litters as described previously [19]. HEK293T cells were purchased from ATCC. Cells were maintained in DMEM (high glucose) supplemented with 3.7 g/L NaHCO_3_, 10% FBS, 100 IU penicillin, 100 mg/mLstreptomycin at 37°C in a humidified incubator containing 5% CO_2_. All cell lines were verified to be free of mycoplasma contamination and authenticated by STR sequencing. PEI at a final concentration of 10 μmol/L was used to transfect HEK293T cells. Total DNA to be transfected for each plate was adjusted to the same amount by using relevant empty vector. Transfected cells were harvested at 24 h after transfection. Lentiviruses, including those for knockdown or stable expression, were packaged in HEK293T cells by transfection using Lipofectamine 2000. At 30 h post transfection, medium (DMEM supplemented with MEM non-essential amino acids; approximately 2 mL) was collected and centrifuged at 5000×*g* for 3 min at room temperature. The supernatant was mixed with 10 μg/mL (final concentration) polybrene, and was added to MEFs or HEK293T cells, followed by centrifuging at 3000×*g* for 30 min at room temperature (spinfection). Cells were incubated for another 24 h (MEFs) or 12 h (HEK293T cells) before further treatments.

To avoid the intrinsic differences of the kinetics of mTORC1 inhibition under glucose starvation between different strains of MEFs and HEK293T cells, all knockout and knockdown cells used were generated from same parental strains. *AMPKβ1*/*2*-DKO HEK293T cells, *AMPKγ1*/*2*/*3*-TKO HEK293T cells, and *AMPKβ1*/*2*-DKO MEFs were generated as described previously [48, 93]. *AMPKα1* and *AMPKα2* in HEK293T cells were knocked down as described previously [20]. The genes (*RRAGA*, *RRAGB*, *TSC2*, *RPTOR*, *RHEBL1*, *AXIN1*, *AXIN2*, *PRKAA1*, *PRKAA2*, *PRKAG1*, *PRKAG2* and *PRKAG3*) were deleted from MEFs or HEK293T cells using the CRISPR-Cas9 system. Note that *Rheb* was not knocked out because we and others [94] found it leads to cell death. Nucleotides were annealed to their complements containing the cloning tag AAAC and inserted into the back-to-back *Bsm*B I restriction sites of lentiCRISPRv2 vector. The sequence for each sgRNA is as follows: 5ʹ-CGTCCGATTCCTAGGGAACC-3ʹ for human *RRAGA*, 5ʹ-ATCTGACTCTGAGAAAACGA-3ʹ for human *RRAGB*, 5ʹ-TCTCATACACTCGAGTGGCG-3ʹ for mouse *TSC2*, 5ʹ-ATACGACCTGCAG ACGTGGA-3ʹ for mouse *RPTOR*, 5ʹ-ATCCTCGGATACCGCTGTGT-3ʹ for human *RHEBL1*, 5ʹ-GGGGTTGACTGGCTCCCGCC-3ʹ for human *AXIN1*, 5ʹ-GTTTC ACCGAAGATGCCCCC-3ʹ for mouse *AXIN1*, 5ʹ-ACACCAGGCGGAACGAAGAT-3ʹ for human *AXIN2*, 5ʹ-GAAGATCGGCCACTACATTC-3ʹ for human *PRKAA1*, 5ʹ-GGGCCGCAATAAAAGATATC-3ʹ for mouse *PRKAA1*, 5ʹ-GGCGG CTCTTTCAGCAGATT-3ʹ for human *PRKAA2*, 5ʹ-GGGAGCCCGTGCGCCGAACA-3ʹ for mouse *PRKAA2*, 5ʹ-AGGGGCGGCACGAACACCAT-3ʹ for mouse *PRKAG1*, 5ʹ-GCGTTTATATGCGATTCATG-3ʹ for mouse *PRKAG2*, and 5ʹ-CTTGACTCCCCGTATGGCAC-3ʹ for mouse *PRKAG3*.

The constructs were then subjected to lentivirus packaging using HEK293T cells that were transfected with 2 µg of DNA in Lipofectamine 2000 transfection reagent per well of a six-well plate. At 30 h post transfection, the virus (approximately 2 mL) was collected for infecting MEFs or HEK293T cells as described above, except cells cultured to 15% confluence which were incubated with virus for 72 h. In particular, for HEK293T cells, a 0.5 mL of fresh DMEM was supplemented to each well after 36 h post infection. When cells approached confluence, they were single-cell sorted into 96-well dishes. Clones were expanded and evaluated for knockout status by sequencing. For glucose starvation, cells were rinsed twice with PBS, and then incubated in glucose-free DMEM supplemented with 10% FBS and 1 mmol/L sodium pyruvate for the desired periods of time at 37°C.

### Immunoblotting

To analyze the levels of p-S6K, p-AMPKα, and p-ACC in MEFs and HEK293T cells, cells grown to 70–80% confluence in a well of a 6-well dish were lysed with 250 μL of ice-cold lysis buffer (20 mmol/L Tris-HCl, pH 7.5, 150 mmol/L NaCl, 1 mmol/L EDTA, 1 mmol/L EGTA, 1% (v/w) Triton X-100, 2.5 mmol/L sodium pyrophosphate, and 1 mmol/L β-glycerophosphate with protease inhibitor cocktail). The lysates were then centrifuged at 20,000×*g* for 10 min at 4°C, and an equal volume of 2× SDS sample buffer was added into the supernatant. Samples were then boiled for 10 min and then directly subjected to immunoblotting.

For immunoblotting, the SDS-polyacrylamide gels were prepared as described previously [93]. Samples of less than 10 μL were loaded into wells, and the electrophoresis was run at 100 V by a Mini-PROTEAN Tetra Electrophoresis Cell (BIO-RAD). In this study, all samples were resolved on 8% resolving gels, except for RAGA, RAGB, AMPKβ1, AMPKβ2, and AMPKγ1, which were resolved on 10% gels, and Rheb and RhebL1, which were resolved on 15% gels. The resolved proteins were then transferred to a PVDF membrane (0.45 μm, cat. IPVH00010, Merck), as described previously [93]. The blotted PVDF membrane was then blocked by 5% (w/v) BSA (for all antibodies against phosphorylated proteins) or 5% (w/v) non-fat milk (for all antibodies against total proteins) dissolved in TBST [40 mmol/L Tris, 275 μmol/L NaCl, 0.2% (v/v) Tween-20, pH 7.6] for 2 h on an orbital shaker at 60 rpm at room temperature, followed by rinsing with TBST (2×, 5 min each). The PVDF membrane was incubated with the desired primary antibody overnight at 4°C on an orbital shaker at 60 rpm, followed by rinsing with TBST (3×, 5 min each, at room temperature), and then the secondary antibodies were added and the membrane incubated for 3 h at room temperature with gentle shaking. The secondary antibody was then removed, and the PVDF membrane was further washed with TBST (3×, 5 min each, at room temperature). PVDF membranes were incubated in ECL mixture (by mixing equal volumes of ECL solution and Peroxide solution for 5 min), then exposed to a medical X-Ray film (FUJIFILM). The films were then developed with an X-OMAT MX Developer and Replenisher in X-OMAT MX Fixer and Replenisher solutions (Carestream) on a Medical X-Ray Processor (Carestream) using Developer (Model 002, Carestream). The developed films were scanned using a Perfection V850 Pro scanner (Epson) using Epson Scan software (v.3.9.3.4) and were cropped using Photoshop 2022 software (Adobe). Levels of total proteins and phosphorylated proteins were analyzed on separate gels, and representative immunoblots are shown. The band intensities on developed films were quantified using ImageJ software (v.1.8.0, National Institutes of Health Freeware).

### Confocal microscopy

For determining the lysosomal localization of AXIN and mTOR, cells grown to 60–80% confluence on coverslips in six-well dishes were fixed for 20 min with 4% (v/v) formaldehyde in PBS at room temperature. The coverslips were rinsed twice with PBS and permeabilized with 0.1% (v/v; for determining AXIN localization) or 0.05% (v/v; for determining mTOR localization) Triton X-100 in PBS for 5 min at 4°C. After rinsing twice with 1 mL of PBS, the coverslips were blocked in 1 mL of 5% NGS (diluted in PBS), and then incubated with primary antibodies diluted in 5% NGS overnight at 4°C. The cells were then rinsed three times with 1 mL of PBS, and then incubated with secondary antibodies for 8 h at 4°C in the dark. Cells were washed for another four times with 1 mL of PBS, and then mounted on slides using ProLong Diamond Antifade Mountant. Confocal microscopic images were taken on a Zeiss Laser Scanning Microscope (LSM) 980 with a 63 × 1.4 NA oil objective. Samples were excited with a diode laser module (BLD-RT 48830 TN01, Lasos) at 488 nm for Alexa Fluor 488 dye (green channel), and with a DPSS laser module (YLK-XT 5948 F01, Lasos) at 594 nm for Alexa Fluor 594 dye (red channel). The parameters, including “PMT voltage”, “Offset”, “Pinhole” and “Gain”, were kept unchanged between each picture taken. The resolution of image is 1024 × 1024 pixels. Images were processed using Zen 3.4 (Zeiss) and formatted by Photoshop 2022 software. All images shown without biological replicates are representative of a minimum of three independent experiments. For quantitative analyses of lysosomal mTOR and AXIN localization percentages (determined by Mander’s overlap coefficient), the number of pixels from the red channel that overlap with pixels from the green channel are divided by the total number of pixels detected in the red channel above the threshold by Zen Black 2012 software. Thresholds were set automatically by the software.

### Statistical analysis

Statistical analyses were performed using Prism 9 (GraphPad Software). For comparisons between two groups, the normality of data was tested by Kolmogorov–Smirnov test, Anderson–Darling test, D’Agostino-Pearson omnibus test, or Shapiro–Wilk test. If the data were not normally distributed (*P* < 0.05), a Mann–Whitney test was used to determine the significance between two groups. If the data were normally distributed, an unpaired two-tailed Student’s *t*-test [for data that had an equal standard deviation (SD), as determined by *F*-test] or an unpaired two-tailed Student’s *t*-test with Welch’s correction (for data that had an unequal SD) was used. For comparisons between multiple groups with one fixed factor, an ordinary one-way ANOVA was used when data were normally distributed, and was followed by Tukey (equal SD, approximately same *n* number between each group), Sidak (equal SD, largely different *n* number between each group), Dunnett (equal SD, specific for comparisons of each group with a single, control group), or Dunnett’s T3 (unequal SD, after corrected by Welch’s correction) multiple comparisons test. When data were not normally distributed, a Kruskal–Wallis test during which Dunn’s multiple comparisons test was used. For comparisons between multiple groups with one fixed factor, an ordinary two-way ANOVA, followed by Tukey’s or Sidak’s multiple comparisons test, was used. The Geisser-Greenhouse’s correction was used before the two-way ANOVA analysis. The adjusted means and SEM were recorded when the analysis met the above standards. Differences were considered significant when *P* < 0.05, or *P* > 0.05 with large differences of observed effects (as suggested in [95, 96]).

## Supplementary Material

load005_suppl_Supplementary_Figures

## Data Availability

The analysis was performed using standard protocols with previously described analysis tools. No custom code was used in this study. Materials and reagents are available upon request.
