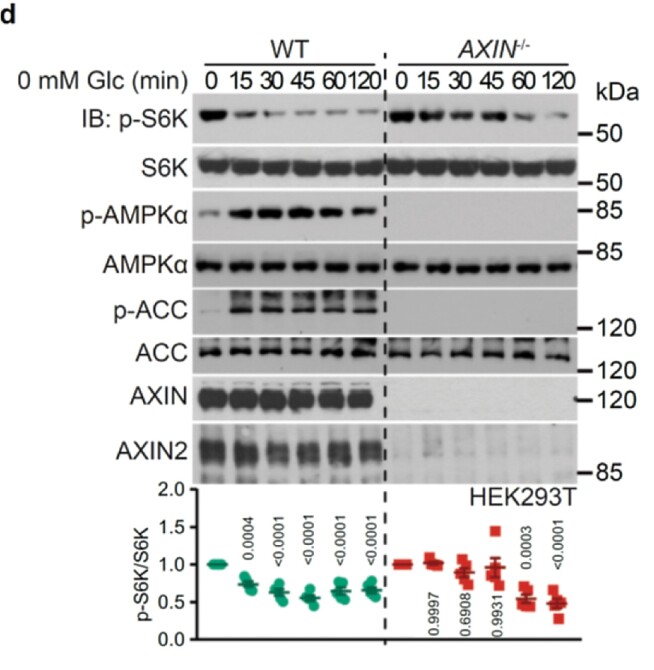# Correction to: Hierarchical inhibition of mTORC1 by glucose starvation-triggered AXIN lysosomal translocation and by AMPK

**DOI:** 10.1093/lifemeta/loaf003

**Published:** 2025-02-12

This is a correction to: Mengqi Li, Xiaoyan Wei, Jinye Xiong, Jin-Wei Feng, Chen-Song Zhang, Sheng-Cai Lin, Hierarchical inhibition of mTORC1 by glucose starvation-triggered AXIN lysosomal translocation and by AMPK, *Life Metabolism*, Volume 2, Issue 3, June 2023, https://doi.org/10.1093/lifemeta/load005.

In Supplementary Figure S1, panel d, the representative image for “A-769662” of the HEK293T cells was wrongly chosen. The statistical analysis data for these images (shown in Figure 1 panel d) remain correct, as they are derived from the appropriate replicates. Supplementary Figure S1 panel d should read:


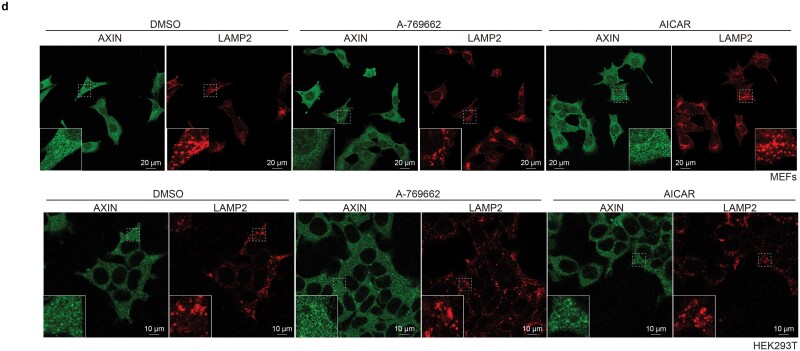


In Figure 2 panel b, the blots of “AMPKα” in the wild-type cells are misplaced. Figure 2 panel b should read:


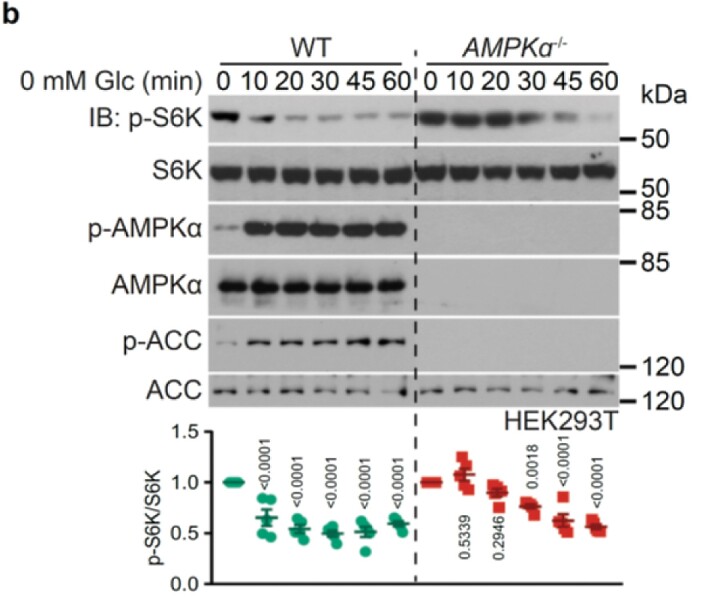


Figure 2 panel c, the blots for “S6K” are misplaced. The statistical analysis data for p-S6K/S6K in this panel remains correct. Figure 2 panel c should read:


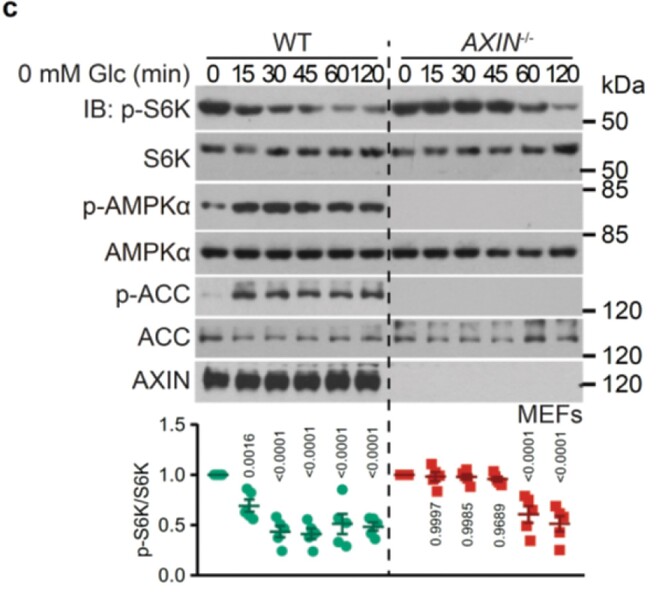


Figure 2 panel d, the blots of “p-ACC” are wrongly placed. Figure 2 panel d should read: