# Supplementary material for: Hierarchical inhibition of mTORC1 by glucose starvation-triggered AXIN lysosomal translocation and by AMPK
Source: Life Metab. 2023 Mar 1;2(3):load005. doi: 10.1093/lifemeta/load005 (PMC11749110; doi:10.1093/lifemeta/load005)
Supplement: load005_suppl_Supplementary_Figures [file load005_suppl_Supplementary_Figures.pdf]

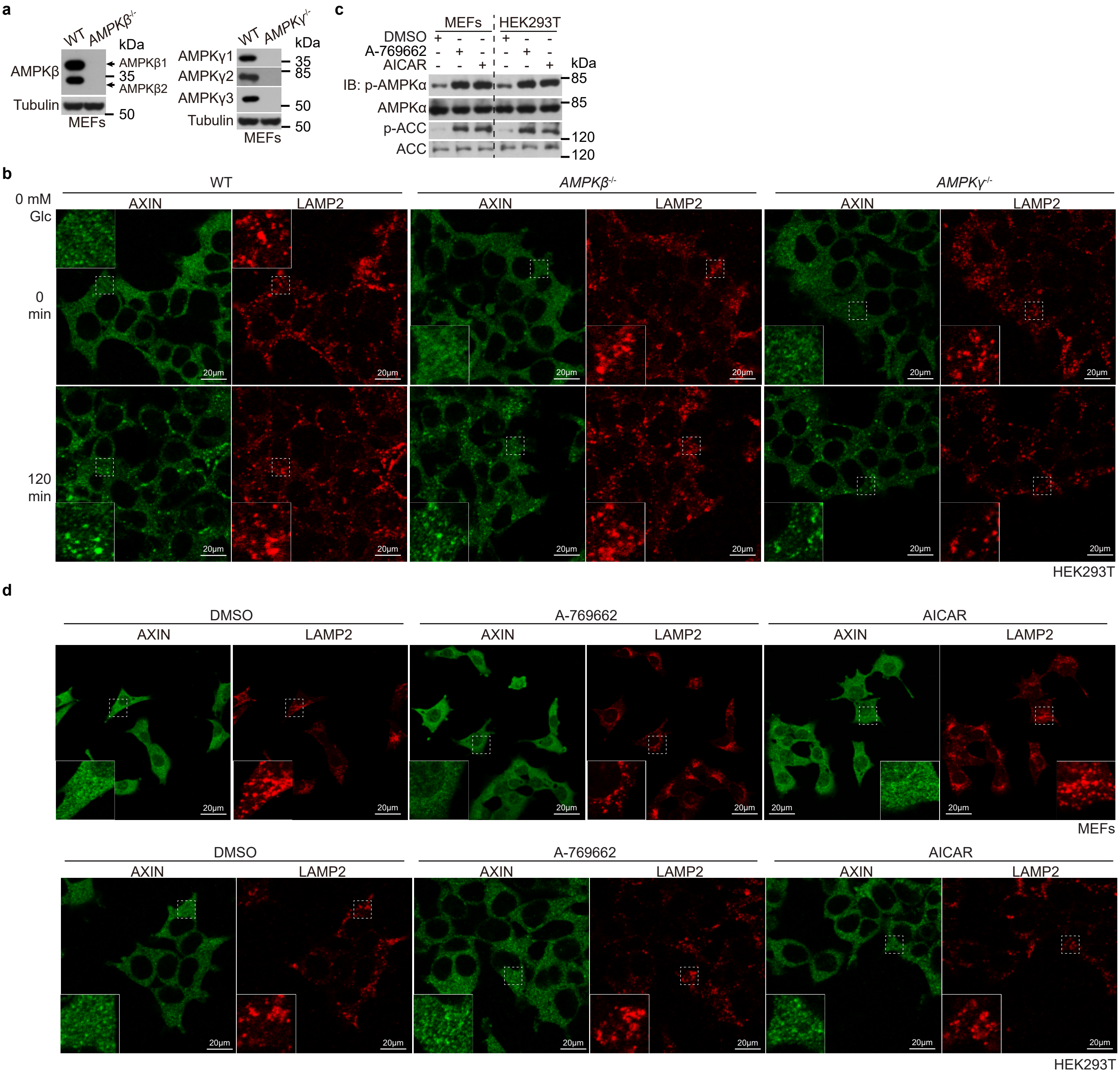

Figure S1 AMPK is not involved in the lysosomal translocation of AXIN in low glucose.
(a) Knockout efficiency of AMPKβ1/2-DKO (left) and AMPKγ1/2/3-TKO (right) MEFs. Cells were lyzed, and the expression levels of each protein were analyzed by immunoblotting.
(b, d) Representative images of Fig. 1c (b) and Fig. 1d (d).
(c) Validation data for the efficiency of AMPK activators on regulating AMPK. MEFs and HEK293T cells were treated 200 μM A-769662, 0.6 mM AICAR, or DMSO control for 2 h, followed by determining p-AMPKα and p-ACC levels.
Experiments in this figure were performed 3 times.

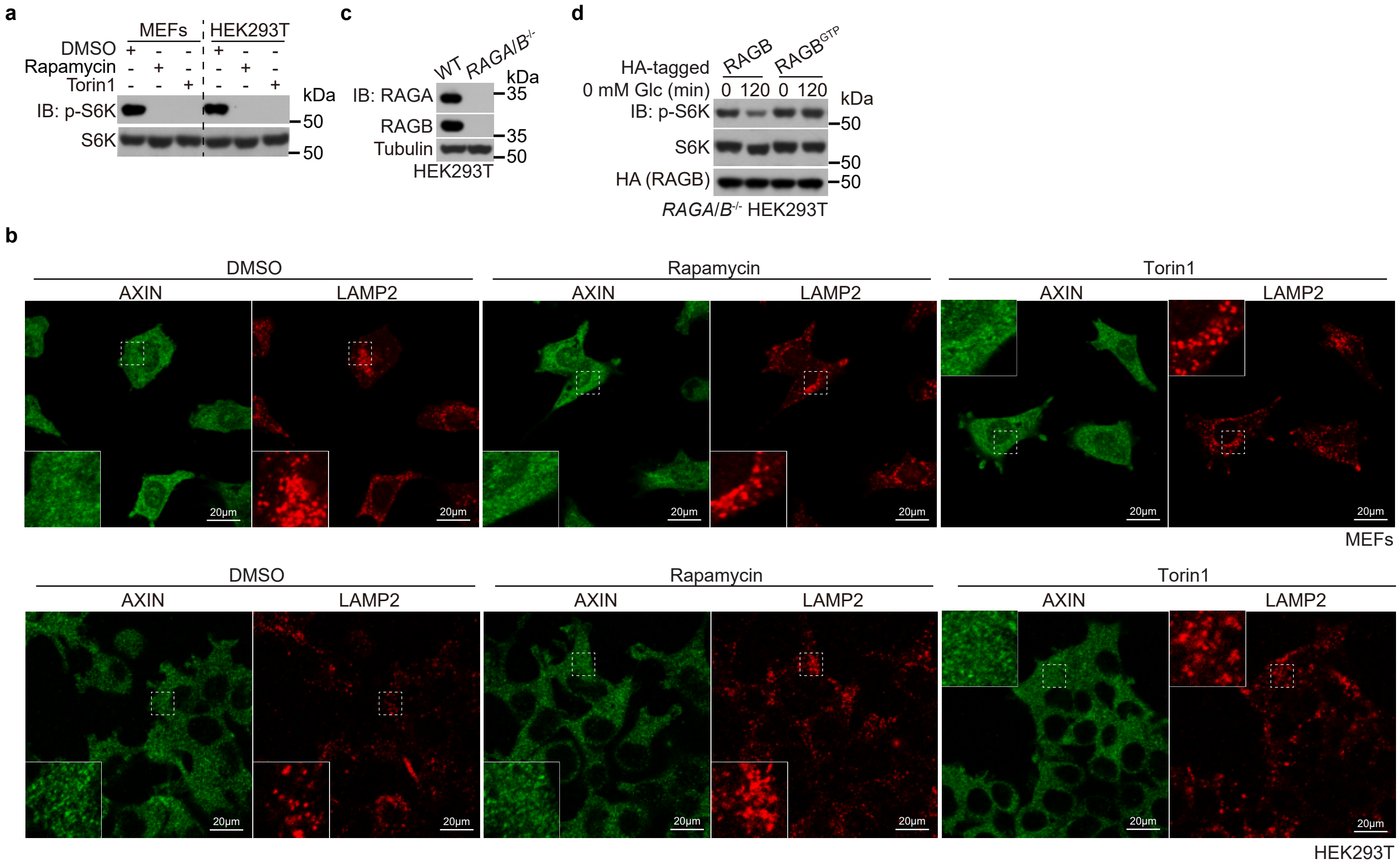

Figure S2 mTORC1 is not involved in the lysosomal translocation of AXIN in low glucose.

(a) Validation data for the efficiency of mTORC1 inhibitors on regulating mTORC1. MEFs and HEK293T cells were treated 100 nM rapamycin, 250 nM Torin1, or DMSO control for 2 h, followed by determining p-S6K and S6K levels.

(b) Representative images of Fig. 1e.

(c) Knockout efficiency of RAGA/B-/- HEK293T cells. Cells were lyzed, and the expression levels of each protein were analyzed by immunoblotting.

(d) Re-introduction of RAGBGTP into RAGA/B-DKO HEK293T cells sufficiently prevents mTORC1 inhibition in low glucose. Cells were starved for glucose for 2 h, followed by determining p-S6K and S6K levels.

Experiments in this figure were performed 3 times.

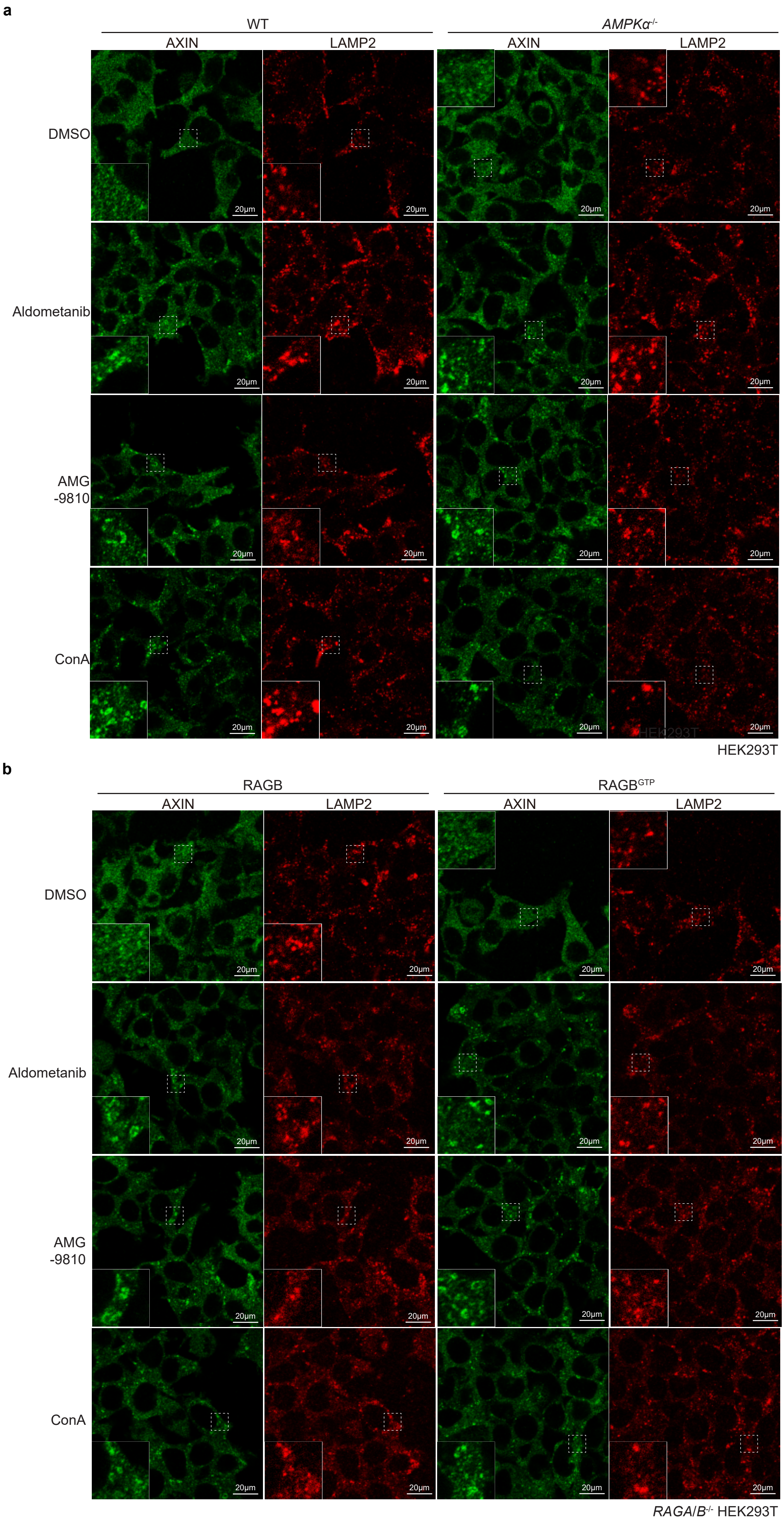

Figure S3 Inhibition of aldolase-TRPV-v-ATPase-Ragulator-RAG axis to mimic the low glucose and trigger AXIN translocation.
HEK293T with AMPKα1/2 knocked out (a), or RAGA/B knocked out with RAGBGTP re-introduced (b), were treated as in Fig. 1g. Representative images are shown.
Experiments in this figure were performed 3 times.

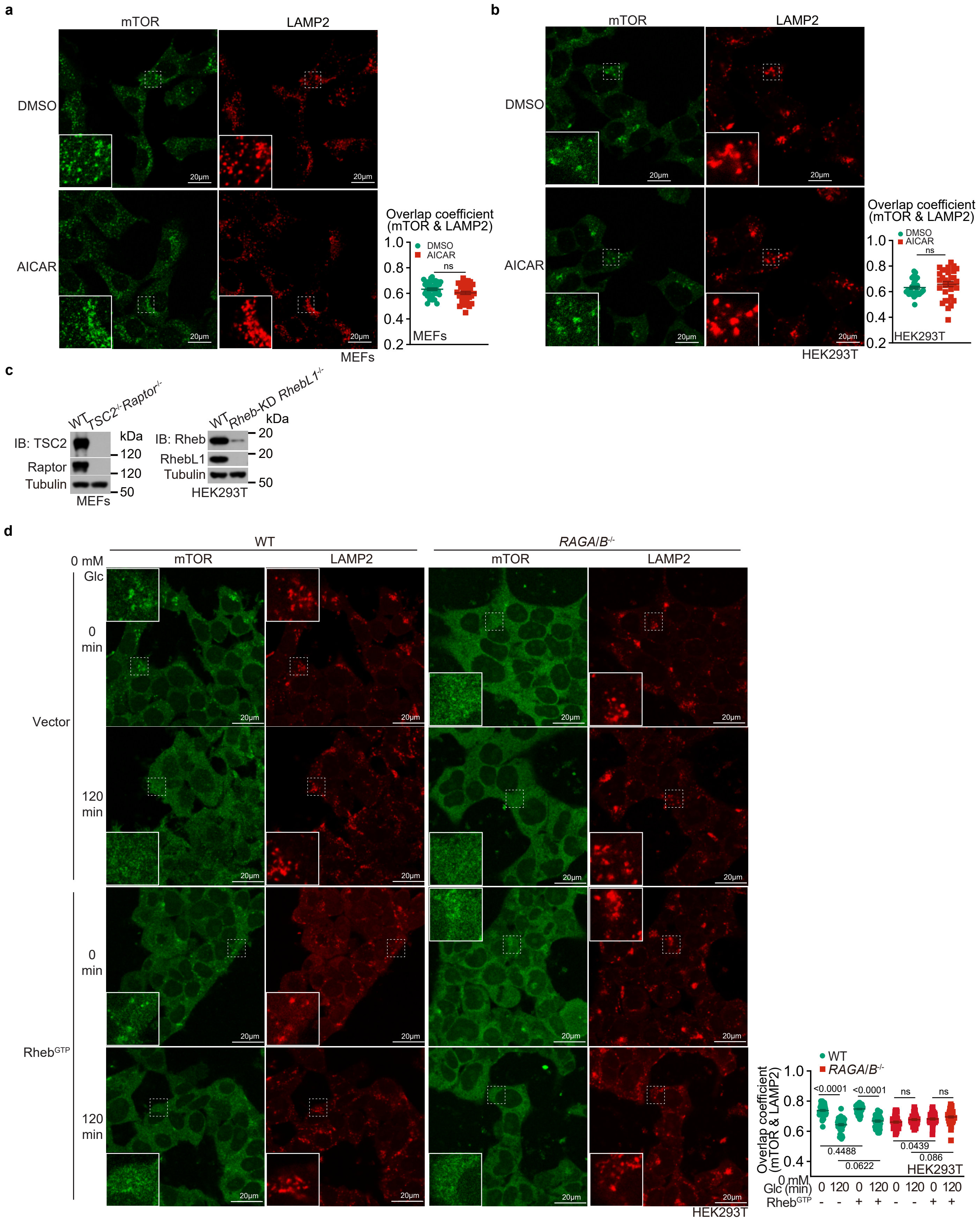

Figure S4 AICAR does not dissociate mTORC1 from the lysosome.

(a, b) MEFs (a) or HEK293T cells (b) were treated with 0.6 mM AICAR for 2 h, followed by determining the co-localization between mTOR and LAMP2. Mander's overlap coefficients are plotted as means ± SEM, n = 34 (a) or 31-32 (b) cells, with P values calculated by two-sided Student's t-test (a), or two-sided Student's t-test with Welch's correction (b).

(c) Knockout and knockdown efficiency of MEFs with knockout of both TSC2 and Raptor (left), or knockdown of Rheb and knockout of RhebL1 (right). Cells were lyzed, and the expression levels of each protein were analyzed by immunoblotting.

(d) Ectopic expression of RhebGTP slightly induces the lysosomal translocation of mTORC1 in low glucose. HEK293T cells and RAGA/B-/- HEK293T cells were transfected with 5 µg of RhebGTP, and were glucose-starved for 2 h, followed by determination of the co-localization between mTOR and LAMP2 by confocal microscopy. Mander's overlap coefficients are plotted as means ± SEM, n = 40-50 cells, with P values calculated by two-way ANOVA, followed by Tukey. Experiments in this figure were performed 3 times.

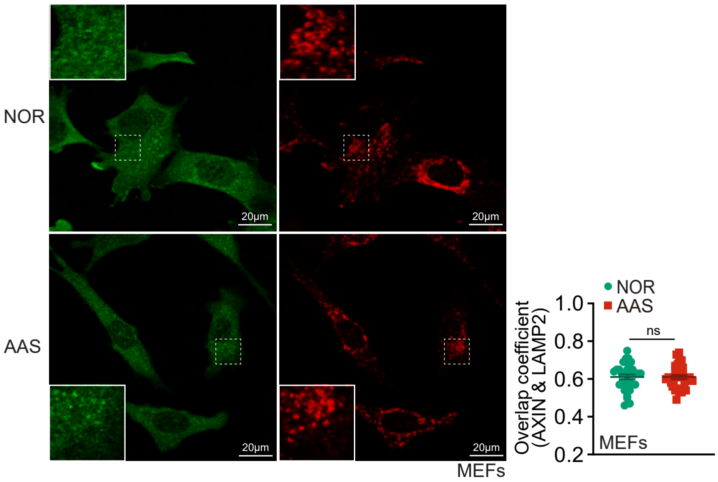

Figure S5 Amino acid starvation does not cause the lysosomal translocation of AXIN.
MEFs were incubated in amino acid-free DMEM for 1 h (AAS), followed by determination of the co-localization between AXIN and LAMP2 by confocal microscopy. Mander's overlap coefficients are plotted as means ± SEM, n = 32 cells, with P values calculated by two-sided Student's t-test.
Experiments in this figure were performed 3 times.
